# Leaf Hydraulic Conductance for a Tank Bromeliad: Axial and Radial Pathways for Moving and Conserving Water

**DOI:** 10.3389/fpls.2013.00078

**Published:** 2013-04-10

**Authors:** Gretchen B. North, Frank H. Lynch, Franklin D. R. Maharaj, Carly A. Phillips, Walter T. Woodside

**Affiliations:** ^1^Department of Biology, Occidental CollegeLos Angeles, CA, USA; ^2^Department of Mathematics, Occidental CollegeLos Angeles, CA, USA

**Keywords:** epiphyte, leaky cable model, mesophyll conductance, monocot leaf, water relations, xylem

## Abstract

Epiphytic plants in the Bromeliaceae known as tank bromeliads essentially lack stems and absorptive roots and instead take up water from reservoirs formed by their overlapping leaf bases. For such plants, leaf hydraulic conductance is plant hydraulic conductance. Their simple strap-shaped leaves and parallel venation make them suitable for modeling leaf hydraulic conductance based on vasculature and other anatomical and morphological traits. Plants of the tank bromeliad *Guzmania lingulata* were investigated in a lowland tropical forest in Costa Rica and a shaded glasshouse in Los Angeles, CA, USA. Stomatal conductance to water vapor and leaf anatomical variables related to hydraulic conductance were measured for both groups. Tracheid diameters and numbers of vascular bundles (veins) were used with the Hagen–Poiseuille equation to calculate axial hydraulic conductance. Measurements of leaf hydraulic conductance using the evaporative flux method were also made for glasshouse plants. Values for axial conductance and leaf hydraulic conductance were used in a model based on leaky cable theory to estimate the conductance of the radial pathway from the vein to the leaf surface and to assess the relative contributions of both axial and radial pathways. In keeping with low stomatal conductance, low stomatal density, low vein density, and narrow tracheid diameters, leaf hydraulic conductance for *G. lingulata* was quite low in comparison with most other angiosperms. Using the predicted axial conductance in the leaky cable model, the radial resistance across the leaf mesophyll was predicted to predominate; lower, more realistic values of axial conductance resulted in predicted radial resistances that were closer to axial resistance in their impact on total leaf resistance. Tracer dyes suggested that water uptake through the tank region of the leaf was not limiting. Both dye movement and the leaky cable model indicated that the leaf blade of *G. lingulata* was structurally and hydraulically well-suited to conserve water.

## Introduction

Tank bromeliads have a captive water supply, held in reservoirs formed by overlapping leaf bases. For these species, the leaf is the organ of both supply and demand: the leaf base absorbs water and nutrients captured in the tank and delivers them to the leaf blade. Thus, the uptake, delivery, and use of water are solely leaf processes; for tank bromeliads, plant hydraulic conductance is the collective hydraulic conductance of its leaves. The hydraulic system for tank bromeliads is simplified not only because absorptive roots and stems are lacking but also because the leaves themselves are classically monocotyledonous: simple, entire, and largely strap-shaped. Water that is absorbed through the leaf base is transpired by the leaf blade after traveling through a vascular system that consists largely of parallel veins. The radial pathways of water into the leaf from the tank and out of the leaf vasculature through the mesophyll and other extravascular tissues are more complex than the axial pathway through the veins, but an overall picture of leaf hydraulic conductance can be developed through a combination of physiological and anatomical measurements and mathematical modeling. The goal of this study is to analyze leaf hydraulics for a tank bromeliad, not only because such plants are hydraulically intriguing and ecologically important in the forests of the Neotropics (Nadkarni, 1984), but also because they represent a sizable group of plants that has been largely neglected with respect to leaf hydraulics: non-grass monocots.

For tank bromeliads as for most plants, water movement through a leaf can be analyzed in terms of the conductances (or inversely, resistances) that determine flow rates, driven by differences in water potential. Assuming that the water potential of the tank contents is higher than that of the leaf blade, water will move up the leaf driven by the lower leaf water potential induced by transpiration. Because leaves of tank bromeliads have one rank of parallel veins roughly centered between the two leaf surfaces (Benzing, 2000), the individual vein conductances can be measured and added together to obtain an approximation of axial conductance for the leaf. Discussions of leaf hydraulic conductance often treat axial and radial conductances as occurring largely in series (Tyree and Yianoulis, 1980; Cochard et al., 2004; Scoffoni et al., 2008; McKown et al., 2010), as makes sense for leaves with a reticulate system of veins, only the smallest of which lose water to the surrounding mesophyll. The analysis applied here to tank bromeliads recognizes that a system composed of parallel veins with few cross-links must be responsible for concurrent axial and radial flow, thus the simple model presented here draws upon equations derived from leaky cable theory (Landsberg and Fowkes, 1978; Frensch and Steudle, 1989; North et al., 2004).

**Figure 1:**
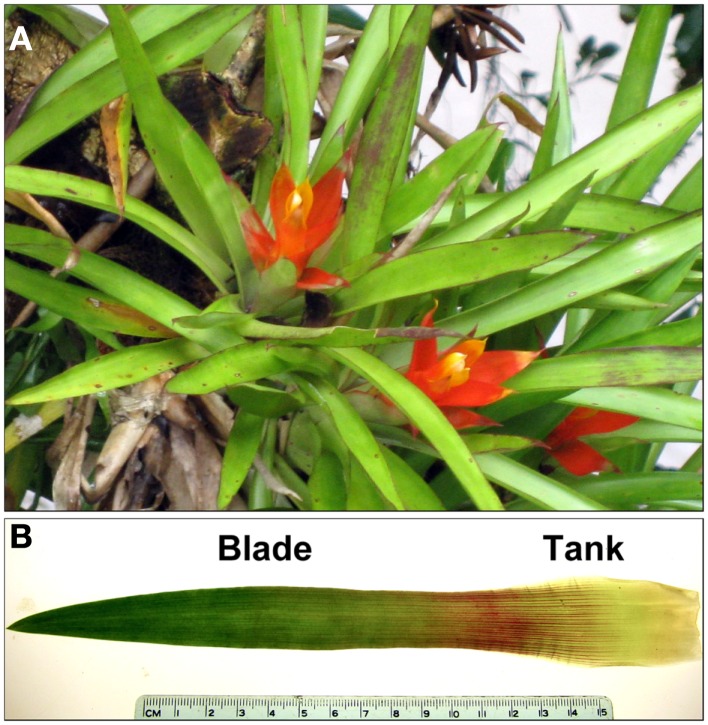
**(A)** Plants of *Guzmania lingulata* in the field at La Selva biological Station, Costa Rica, and **(B)** a single leaf of *G. lingulata* labeled with the two leaf regions examined.

The tank bromeliad *Guzmania lingulata* (Figure 1A) was chosen for study because of its simple and regular leaf shape, its relatively widespread occurrence in the Neotropics (Griffiths and Maxwell, 1999), and its commercial availability. Tanks in this species are not central but axillary, at the cupped base of each leaf. Occurring at various positions within the forest canopy, *G. lingulata* is considered shade-tolerant, although a relatively high light-saturation point of 400–500 μmol m^−2^ s^−1^ was reported for plants in Trinidad (Griffiths et al., 1986). Physiological and anatomical traits related to leaf hydraulic conductance were investigated for plants growing in the forest understory at La Selva Biological Station in Costa Rica and compared with traits of plants grown in a glasshouse at Occidental College in Los Angeles, CA. Previous measurements of gas exchange for *G. lingulata* as well as preliminary observations of its physiological ecology and leaf anatomy led to the prediction that leaf hydraulic conductance would be lower than for most other angiosperms. Generally speaking, conservative water use for tank bromeliads is to be expected, given their lack of access to soil water, low relative growth rates, and relatively long leaf lifespans (Schmidt and Zotz, 2002; Meisner and Zotz, 2012). No prediction was made as to the relative contributions of axial and radial conductances to total leaf hydraulic conductance, given the wide range for other species reported in the literature. We used a leaky cable model to assess the components of leaf hydraulic conductance in the context of xylem structure and other anatomical variables for leaves of *G. lingulata*. Such a model, though subject to further refinement, can help inform our understanding of what structures and pathways pose the greatest resistance to water movement through leaves.

## Materials and Methods

### Plant material and environmental measurements: La Selva, Costa Rica

Leaf sampling and field observations were done in June 2012 for plants of *G. lingulata* (L.) Mez (Bromeliaceae) growing in wet, lowland tropical forest at La Selva Biological Station (84°00′12″ W, 10°25′52″ N) in northeastern Costa Rica. Leaves for anatomical analysis were collected from plants growing on host trees (or stumps) at heights ranging from 0.3 to 2.0 m from the ground. The fourth leaf from the center of each plant was removed and photographed for leaf area, which was calculated using ImageJ (freeware available)^1^. Sections from the leaf blade and the tank region (Figure 1B) were fixed in formalin: acetic acid: alcohol for 2 days and then stored in 40% ethanol for later analysis at Occidental College. Epidermal impressions for measurement of stomatal and trichome densities were made using clear nail polish, removed when dry with cellophane tape and affixed to microslides.

Light environments for plants at La Selva were characterized as % total transmitted light, calculated from photographs taken with a digital camera with a fisheye lens and processed using the computer program Gap Light Analyzer version 2 (Simon Fraser University, BC, Canada). Instantaneous measurements of photosynthetically active radiation (PAR; μmol m^−2^ s^−1^) were made with a handheld quantum meter (Apogee Instruments, Logan, UT, USA). Stomatal conductance to water vapor, *g*_s_ (mmol m^−2^ s^−1^) was measured at midday using a steady-state porometer (SC-1, Decagon Devices, Inc., Pullman, WA, USA), calibrated before use and read in manual mode, suitable for low values of *g*_s_.

### Plant material and environmental measurements: Glasshouse, Los Angeles, USA

Plants of *G. lingulata* were purchased from a local southern California nursery and from the mail-order supplier Tropiflora^2^. All plants were allowed to flower for positive species identification, and were grown in a shaded glasshouse at Occidental College, Los Angeles, CA, USA (34° 7′39″ N, 118°12′37″ W) for at least 14 days before measurements were made. Light levels in the glasshouse averaged 20% of ambient solar radiation; daily average maximum/minimum temperatures were 28/16°C, with an annual maximum/minimum of about 35/10°C. Stomatal conductance to water vapor was measured at midday on plants that were briefly positioned in ambient conditions outside the glasshouse (otherwise, values of *g*_s_ were too low to measure). Leaves were collected and processed for anatomical analysis as in the field at La Selva.

### Leaf anatomy and calculated leaf axial hydraulic conductance (*K*_x_)

Cross-sections from the mid-lamina (blade) and tank regions were made by drawing a razor blade across the width of the leaf, using another razor blade as a straight-edge. Sections were stained with 0.1% (w/w) toluidine blue O in phosphate buffer for general anatomical features and examined using a Nikon Eclipse ME 600 light microscope (Nikon Instruments, Inc., Melville, NY, USA) at magnifications of 40–1000×. To detect suberin and cutin, sections were stained with 0.1% (w/w) Sudan red 7B in 70% ethanol; for lignin, sections were stained with 0.5% (w/w) phloroglucinol in water followed by 20% HCl. Photographs were made using a Spot RT Color digital camera (Diagnostic Instruments, Inc., Sterling Heights, MI, USA), and all measurements were made using ImageJ on calibrated images.

**Figure 2:**
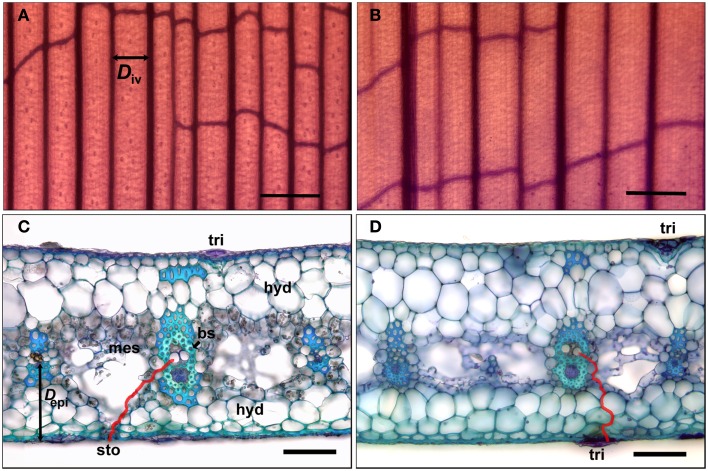
**Sections of leaves of *G. lingulata* used to measure anatomical traits**. Leaf clearings of the leaf blade **(A)** and tank region **(B)** used to determine interveinal distance (*D*_iv_; black arrow); scale bars in **(A)** and **(B)** = 500 μm. Cross-sections of the leaf blade **(C)** and tank region **(D)**; red lines indicate pathways for water between vein and abaxial surface (red line in **(C)** used to calculate *D*_mes;_ black arrow indicates distance between vein and epidermis *D*_epi_); scale bars in **(C)** and **(D)** = 50 μm. Abbreviations: bundle sheath cells (bs), hydrenchyma (hyd), mesophyll (mes), stomate (sto), and trichome (tri).

Vein (vascular bundle) density *D*_v_ (mm mm^−2^) and the distance between veins *D*_iv_ (μm) were measured using leaf clearings (Ruzin, 1999) made from blade and tank regions (Figures 2A,B). Vein lengths for *D*_v_ were measured using straight line and freehand tools in ImageJ, and *D*_iv_ was measured for main veins only, from the center of each vein to the next. The distance from the center of main veins to the epidermis *D*_epi_ (μm) was measured from leaf cross-sections, which were also used for measurements of *D*_mes_ (μm), the likely pathway for water from the tracheids in a vein across the mesophyll and to the guard cells of the stomates. Specifically, *D*_mes_ was measured from freehand tracings (red line, Figure 2C) using ImageJ, assuming that water exits the bundle sheath cells through the symplastic pathway (plasmodesmata) and then travels in the apoplast until exiting between the guard cells (Brodribb et al., 2007). For the tank region, *D*_mes_ was measured from the center of a vein to the closest abaxial trichome (Figure 2D), the epidermal feature associated with water uptake (Benzing, 2000). Pathways for water movement through the leaf blade were investigated by cutting leaves under water, immersing the cut end in 0.1% (w/w) aqueous basic fuchsin (Chatelet et al., 2008), allowing the leaf to transpire for 1-2 h, and examining cross-sections cut above the level of the dye under the microscope (bright field illumination). Radial water uptake through the tank region was investigated by removing a leaf, sealing its cut end at the base of the tank region with dental impression material (Reprosil Light Body; Dentsply International, Woodbridge, ON, Canada), immersing 20 mm of the tank region in a 0.1% (w/w) aqueous solution of sulforhodamine G for 2 h, and examining cross-sections cut above the level of the dye under the microscope (epifluorescence). Both basic fuchsin (Chatelet et al., 2008) and sulforhodamine G ostensibly stain the apoplast (Canny, 1986).

To calculate the maximum theoretical axial (xylem) hydraulic conductance for a leaf *K*_x_ (m^4^ s^−1^ MPa^−1^), the number of veins per mm was measured for leaf cross-sections viewed at 40× and multiplied by the leaf width to determine the number of veins across the leaf. For *G. lingulata* as for most bromeliads (Benzing, 2000) main veins are parallel and in one rank; secondary lateral veins were relatively infrequent and were not considered in the calculation of *K*_x_. Tracheid diameters in at least 20 veins were measured per leaf, treating the tracheids as circular in outline, with the diameter equaling that of the largest circle to be circumscribed within the tracheid. While this is an approximation, the largest tracheids were fairly circular; note that this treatment may overestimate the true conductance due to the presence of tracheid end walls and resistance associated with pit membranes (Lewis and Boose, 1995). The diameters *d* (m) were used in the Hagen–Poiseuille equation to calculate *K*_x_ (Nobel, 2009):

(1) $$K_{\text{x}}=\overset{N}{\underset{i=1}{\sum }}\frac{\pi d_{i}^{4}}{128\eta }$$

where *N* is the number of tracheids in each vein multiplied by the number of veins in the leaf, and η is the viscosity of water (1.0021 × 10^−9^ MPa s at 20°C).

### Measurement of leaf hydraulic conductance (*K*_leaf_)

Total leaf hydraulic conductance *K*_leaf_ (m^3^ mm^−2^ s^−1^ MPa^−1^ or mmol m^−2^ s^−1^ MPa^−1^) was measured for plants grown in the glasshouse, using the evaporative flux method (Sack et al., 2002). The fourth leaf from the center of the plant was selected and removed from the plant by a gentle tug, and the base was immersed in distilled water and cut again right above the tank region (Figure 1B). Because the tank region was not included, *K*_leaf_ as measured here was for the leaf blade only, with water taken up through the xylem in the cut leaf base (roughly analogous to a cut petiole), not absorbed through the leaf surface. The leaf was placed in a plastic vial just large enough to accommodate the leaf width (30 mm in diameter) containing distilled water that had been filtered (pore size 0.2 μm) and stirred under vacuum overnight to remove air bubbles. The water covered approximately 5 mm at the base of the leaf blade, and the remainder of the blade stood upright with most of the leaf surface outside the vial. The vial containing the leaf was placed on a balance capable of reading 0.1 mg, interfaced with a computer program to record weight every 30 s. The leaf was illuminated by a red, blue, and white LED lamp that produced 800–1000 μmol m^−2^ s^−1^ of PAR at the top of the leaf, measured with the Apogee quantum meter. Leaf temperature, monitored with an infrared thermometer, averaged 23°C, and air temperature averaged 20–22°C.

When weight loss readings stabilized, typically within 10 min, weight loss was recorded for 10 min, and an average value for volumetric flow (m^3^ s^−1^) was calculated. The leaf was then removed from the balance and leaf water potential Ψ_leaf_ (MPa), the driving force for water uptake, was measured using a pressure chamber (PMS Instruments, Portland, OR, USA). To correct for evaporation, weight loss of a vial of water without a leaf was measured under the same conditions, and the average value (usually less than 10% of weight loss with a leaf) was subtracted from the value with a leaf. Leaf length and leaf area were measured from digital photographs, using ImageJ.

### Model to calculate leaf radial hydraulic conductance

Using leaf dimensions, measured values for *K*_leaf_, and calculated values for *K*_x_, *K*_r_ (radial conductance; m^3^ m^−2^ MPa^−1^, or mmol m^−2^ s^−1^ MPa^−1^) was calculated using a model based on leaky cable theory as developed by Landsberg and Fowkes (1978) to analyze water movement through roots. The model assumes that along the length of the leaf *l* (m) there is an axial flux *J*_x_ (m^3^ s^−1^) through the xylem driven by a gradient in water potential and a radial flux *J*_r_ (m^3^ m^−2^s^−1^) through leaf tissues external to the xylem driven by a potential difference between the source of water at the base of the leaf (for *G. lingulata*, in the tank or vial) and at the point of evaporation near the leaf surface. Water flows through an axial resistance *R*_x_ (inverse of axial conductance *K*_x_; MPa s m^−4^) in the xylem and through a radial resistance *R*_r_ (inverse of radial conductance *K*_r_; MPa s m^−1^) between the xylem and the leaf surface. Because *G. lingulata*, like all tank bromeliads and most shade-tolerant C_3_ species, has stomates only on the abaxial surface of the leaf, the model considers *J*_r_ to occur in only one direction, toward the abaxial epidermis.

In the model the fluxes satisfy

(2) $$-R_{\text{x}}J_{\text{x}}=\frac{d\Psi }{dz}$$

and

(3) $$-R_{\text{r}}J_{\text{r}}=\Psi -\Psi_{\text{mes}}$$

where Ψ_mes_ is the water potential of the mesophyll at the site of evaporation (estimated as the leaf water potential Ψ_leaf_ (Martre et al., 2001; Nobel, 2009). Conservation of flow in a small region of the leaf [*z*, *z* + Δ*z*] leads to

(4) $$J_{\text{x}}(z)=J_{\text{x}}(z+\text{Δ}z)+w\text{Δ}zJ_{\text{r}}(\bar{z})$$

for some increment of length $\bar{z}\in \left[z,\;z+\Delta z\right]$, and *w* is the width of the leaf, given that flow occurs across only half the perimeter, which is approximately 2*w*, leaf thickness being negligible. In the limit as Δ*z* → 0, the axial and radial fluxes satisfy

(5) $$\frac{dJ_{\text{x}}}{dz}=-wJ_{\text{r}}.$$

Therefore,

(6) $$\frac{d^{2}\Psi }{dz^{2}}=-R_{\text{x}}\frac{dJ_{\text{x}}}{dz}=-R_{\text{x}}\left(wJ_{\text{r}}\right)=\frac{wR_{\text{x}}}{R_{\text{r}}}\left(\Psi -\Psi_{\text{mes}}\right).$$

If the water potential at *z* = 0 (the base of the leaf) is known and the flux at *z* = *l* (the tip of the leaf)is zero, the boundary value problem is

(7) $$\begin{array}{rcl} \frac{d^{2}\Psi }{dz^{2}}-\alpha^{2}\Psi & =-\alpha^{2}\Psi_{\text{mes}}, & \end{array}$$

(8) $$\begin{array}{rcl} \Psi \left(0\right) & =\Psi_{0} & \end{array}$$

and

(9) $$\frac{d\Psi }{dz}\left(l\right)=0,$$

where α^2^ = *wR*_x_/*R*_r_. The solution to this system is written as

(10) $$\Psi \left(z\right)=\Psi_{\text{mes}}=c_{1}\cosh\alpha \left(l-z\right)+c_{2}\;\sinh\alpha \left(l-z\right).$$

To satisfy the boundary conditions it must be that *c*_2_ = 0 and *c*_1_ = (Ψ_0_ − Ψ_mes_)/cosh α*l*. The solution is

(11) $$\Psi \left(z\right)=\Psi_{\text{mes}}+\frac{\Psi_{0}-\Psi_{\text{mes}}}{\cosh\alpha l}\cosh\alpha \left(l-z\right)$$

Again by conservation, the total flux *J*_leaf_ is equal to the axial flux at the base of the leaf, *J*_leaf_ = *J*_x_(*l*), so that

(12) $$-R_{\text{x}}J_{\text{leaf}}=R_{\text{x}}J_{\text{x}}\left(l\right)=\alpha \left(\Psi_{0}-\Psi_{\text{mes}}\right)\tanh\alpha l.$$

Since the effective total resistance satisfies −*R*_leaf_*J*_leaf_ = Ψ_0_ − Ψ_mes_, it is related to the axial and radial resistances by *R*_x_ = α*R*_leaf_ tanh α*l*, or

(13) $$R_{\text{leaf}}=\frac{\Psi_{0}-\Psi_{\text{mes}}}{J_{\text{leaf}}}=\frac{R_{\text{x}}}{\alpha \tanh\alpha l}=\frac{R_{\text{r}}}{w}\;\frac{R_{\text{r}}}{l\tanh\alpha l}=\frac{R_{\text{r}}}{wl^{\prime }}$$

Rearranging and substituting *K*_r_ for 1/*R*_r_ and *K*_leaf_ for 1/*R*_leaf_ gives

(14) $$K_{\text{r}}=\frac{K_{\text{leaf}}\alpha l}{\tanh\alpha l}$$

where $\text{α}=\frac{\sqrt{wK_{\text{r}}}}{K_{\text{x}}}$ and $l^{\prime }=l\frac{\tanh\text{α}l}{\text{α}l}$ is the effective length of the leaf. That is, *l*′ is the length of the leaf along which the demand for water can be met by the supply. Two simplifying assumptions of the model, that neither Ψ_mes_ nor *R*_r_ varies along the length of the leaf blade, will be considered in the Section “Discussion.”

A computer program using these equations to solve for *K*_r_ and *l*′ required as inputs leaf width (*w*), leaf length (*l*), *K*_x_ calculated using Eq. 1, *K*_leaf_ measured by the evaporative flux method, and Ψ_leaf_, which was assigned a value 0.05 MPa lower than the value obtained after measurements of *K*_leaf_. The mean Ψ_leaf_ at the cut end of the leaf blade was −0.43 ± 0.045 MPa (*N* = 8). The program set *K*_r_ initially equal to *K*_leaf_ and obtained a solution for *K*_r_ by iteration. Units for *K*_leaf_, *K*_x_, and *K*_r_ were normalized by leaf area and leaf length for the sake of comparison and converted to mmol m^−2^ s^−1^ MPa^−1^.

Statistical analyses were performed using Sigmastat 3.5 and SigmaPlot 11.0 (Systat Software Inc., San Jose, CA, USA), all with α set at 0.05; data are reported as means ± 1 SE. For plants in the field and in the glasshouse, *N* = 7 unless otherwise noted.

## Results

### Stomatal conductance (*g*_*s*_); stomatal and trichome densities

Despite the variability of light conditions in the forest understory at La Selva due to changes in cloud cover and sunflecks, mean values for midday PAR were similar between the two sites, as were maximum values of PAR at the time measurements of *g*_s_ were made (Table 1). Values for *g*_s_ in both field and glasshouse were frequently too low to measure, but mean values for actively transpiring plants did not differ for plants at the two sites (Table 1).

**Table 1:** **Stomatal conductance (*g*_s_) and light environments for *G. lingulata***.

Site	*g*_s_ (mmol m^−2^ s^−1^)	PAR mean midday; (μmol m^−2^ s^−1^)	% Transmitted light (of total)
La Selva	24.7 ± 1.6	253 ± 143	29.3 ± 3.1
Glasshouse, Los Angeles	23.3 ± 3.5	159 ± 12	–

*Data are means ± 1 SE; *N* = 7 plants from each site*.

Similarly, plants at La Selva did not differ from those in the glasshouse with respect to most stomatal and trichome densities (Table 2).

**Table 2:** **Stomatal and trichome densities for leaves of *G. lingulata***.

Site, leaf region and surface		Stomatal density (mm^−2^)	Trichome density (mm^−2^)
**LA SELVA**			
Blade	Abaxial	23.7 ± 1.5	12.3 + 1.3
	Adaxial	0	9.1 ± 1.2
Tank	Abaxial	0.9 ± 0.2	42.8 ± 1.9
	Adaxial	0	49.8 ± 2.8
**GLASSHOUSE**			
Blade	Abaxial	25.9 ± 1.4	32.4 ± 0.7
	Adaxial	0	14.4 ± 2.3
Tank	Abaxial	17.6 ± 1.6	40.1 ± 2.0
	Adaxial	0	43.1 ± 2.2

*Data are means ± 1 SE.; *N* = 7 plants from each site*.

For both groups, stomates were absent from the blade adaxial surface and were reduced in number on both leaf surfaces of the tank region. Plants at La Selva had a significantly higher stomatal density on the abaxial surface than did glasshouse plants (*t*-test; *P* < 0.001), perhaps reflecting greater variability in depth of tank water in the field. Trichome densities were higher in tank than in blade regions and were similar for plants from the two sites, although plants from La Selva had a higher trichome density on the abaxial surface of the blade than did glasshouse plants (*P* < 0.001).

### Leaf anatomical traits

For all anatomical traits examined, there were no significant differences between plants at the two sites (Table 3). However, several traits differed between leaf blade and tank regions within both groups. Mean tracheid diameter and mean maximum tracheid diameter were higher in the tank region, but only significantly so for plants at La Selva (Table 3). For leaves from glasshouse plants, tracheid diameters were slightly but not significantly smaller near the tip of the leaf than at mid-blade (*N* = 3; *t*-test; *P* = 0.278).

**Table 3:** **Anatomical traits for leaves of *G. lingulata***.

Site leaf region	Tracheid diameter (μm)	Max. tracheid diameter (μm)	*D*_v_ (mm mm^−2^)	*D*_iv_ (μm)	*D*_epi_ (μm)	*D*_mes_ (μm)
**LA SELVA**						
Blade	6.57 ± 0.11	11.72 ± 0.34	4.99 ± 0.17	250.8 ± 10.6	126.2 ± 6.37	180.9 ± 7.5
Tank	8.02 ± 0.11	13.19 ± 0.13	3.66 ± 0.06	333.2 ± 14.8	192.0 ± 2.92	206.4 ± 18.1
**GLASSHOUSE**						
Blade	6.38 ± 0.30	11.79 ± 0.67	4.73 ± 0.04	247.4 ± 6.9	117.0 ± 3.23	181.7 ± 6.3

*Data are means ± 1 SE.; *N* = 7 plants from each site*.

Maximum tracheid diameters were measured in approximately every fourth vein (shown in Figure 2A), with tracheids in the intervening veins having smaller average diameters. As calculated from leaf clearings (Figures 2A,B), plants at both sites had significantly higher vein densities (*D*_v_) and lower interveinal distances (*D*_iv_) in the leaf blade than in the tank region (*t*-test; *P* < 0.001 for both traits). Similarly, the linear distance from the middle of a vein to the abaxial epidermis (*D*_epi_; Figure 2C) was smaller in the leaf blade (Figure 2C) than in the tank region (Figure 2D; Table 3).

The red line in Figure 2C indicates *D*_mes_ for the leaf blade, indicating a potential pathway for water from a tracheid through plasmodesmata in the lignified and suberized bundle sheath cells, and then through the cell walls (the apoplast) of the mesophyll and the water storage tissue (hydrenchyma) before reaching the guard cells of the stomate. The primary photosynthetic region of the mesophyll surrounded the veins in the center of the blade (Figure 2C), with irregularly shaped (also called stellate) mesophyll cells forming a loose mesh above the substomatal chamber. For the tank region, the red line indicates *D*_mes_ as the pathway of water from the nearest trichome to a tracheid (Figure 2D). *D*_mes_ tended to be greater for the tank region than for the leaf blade, although differences were not significant due to large variances (Table 3). The intercellular meshwork in the tank region formed lacunae or regions of aerenchyma that did not usually extend to the epidermis (Figure 2D). As indicated by staining with sudan dye (not shown), both guard cells and the cells that flank the central (stalk) cells in the trichome had suberized or cutinized cell walls.

### Tracer dye movement

**Figure 3:**
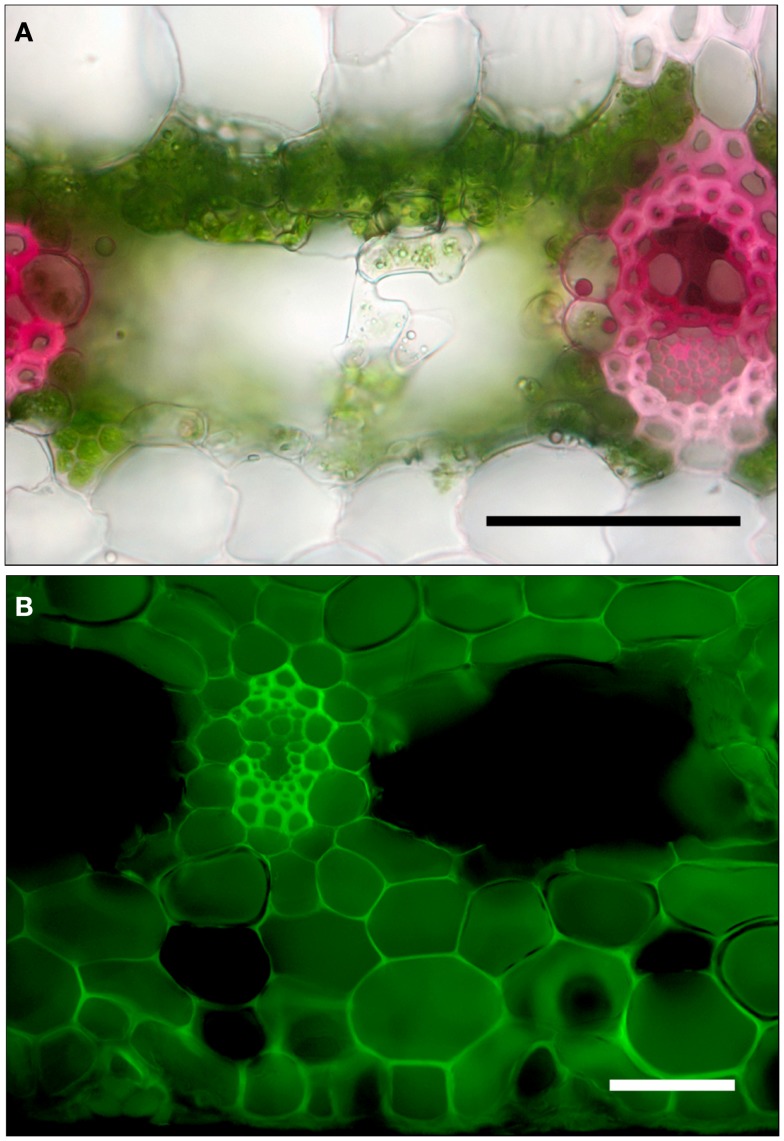
**Cross-sections of leaves of *G. lingulata***. **(A)** Leaf blade after cut end was immersed in 0.1% basic fuchsin dye for 1 h. **(B)** Tank region, after leaf was cut, sealed at the cut end, and immersed in 0.1% sulforhodamine G for 1 h; scale bars in **(A)** and **(B)** = 50 μm.

When leaves were cut at the base of the blade, placed in a solution of basic fuchsin dye, and allowed to transpire under lights, the dye traveled about 2 cm h^−1^. In leaf cross-sections examined under the microscope, dye was strongly concentrated in most tracheids and the cell walls of the bundle sheath cells (Figure 3A). Similar results were obtained when the cut end of the leaf blade was immersed in sulforhodamine G (not shown). When leaves were cut at the base of the tank region, sealed, and placed in sulforhodamine G for 2 h, the dye was apparent not only in the cell walls but also in the cytoplasm or vacuole (Figure 3B). Sections were covered with immersion oil immediately after being cut to prevent diffusion of the stain, and no post-cutting diffusion was observed in internal air spaces. In the tank region, water apparently moved inward from absorbing trichomes in both adaxial and abaxial surfaces (Figures 2D and 3B).

### Leaf hydraulic conductance

**Figure 4:**
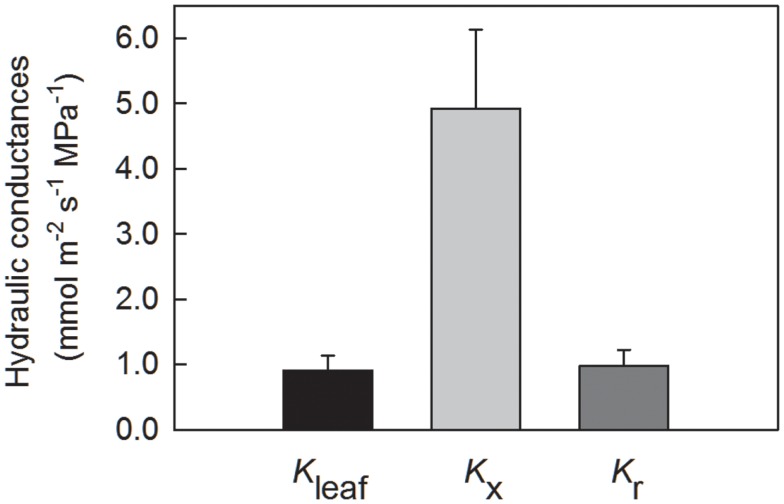
**Leaf hydraulic conductances for *G. lingulata*, normalized by leaf blade area and leaf length**. Data are means ± 1 SE; *N* = 7 plants.

Leaf hydraulic conductance *K*_leaf_ for leaf blades from glasshouse plants was 0.901 ± 0.23 mmol m^−2^ s^−1^ (*N* = 7; Figure 4), as measured using the evaporative flux method. Axial hydraulic conductance *K*_x_, as calculated using the number and diameters of tracheids in Eq. 1, was 4.42 × 10^−11^ ± 1.22 × 10^−11^ m^4^ s^−1^ MPa^−1^. For plants at La Selva, *K*_x_ calculated using the Hagen–Poiseuille equation (Eq. 1) was 5.15 × 10^−11^ ± 1.07 × 10^−11^, not significantly different from glasshouse plants (*t*-test; *P* = 0.66). Values of *K*_x_ were corrected by leaf length and expressed on a leaf area basis for the sake of comparison with *K*_leaf_ (Figure 4). Radial hydraulic conductance *L*_r_ (from the xylem to the abaxial leaf surface) for leaf blades from glasshouse plants was calculated using values for *K*_leaf_ and *K*_x_ plus leaf dimensions and leaf water potential in Eqs 13 and 14 (Figure 4). Radial conductance was 7% greater than *K*_leaf_ yet approximately five times smaller than *K*_x_. In other words, the axial pathway through the xylem calculated using Eq. 1 represented the largest conductance (and therefore the smallest resistance) of the three.

**Figure 5:**
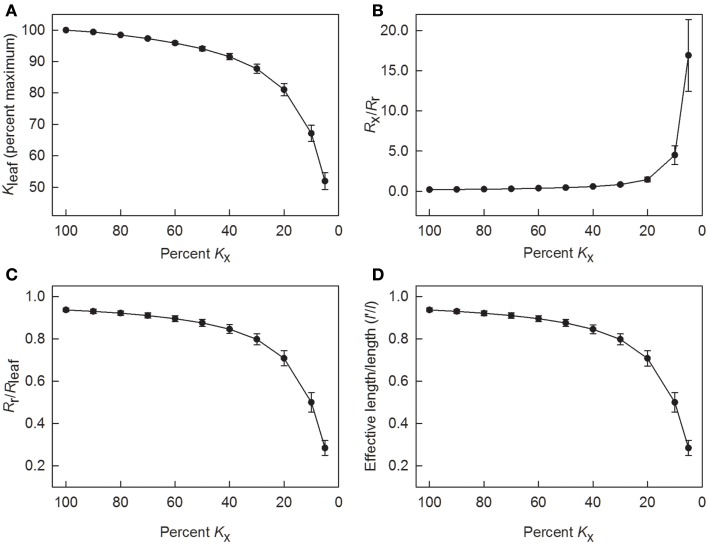
**Results of using the leaky cable model to calculate radial conductance (*K*_r_) and to determine values of leaf hydraulic conductance (*K*_leaf_), as well as resistances *R*_r_, *R*_x_, and *R*_leaf_ as axial conductance (*K*_x_) was reduced in steps**. **(A)** changes in axial conductance as a percent of leaf hydraulic conductance; **(B)** changes in the ratio of axial to radial resistance; **(C)** changes in the ratio of radial resistance to total leaf resistance; and **(D)** changes in the ratio of the effective length for leaf water uptake (*l*′) to the actual leaf length (*l*). Data are means ± 1 SE; *N* = 7 plants.

Because tracheids are not ideal capillaries due primarily to the presence of end walls and the resistance of the pit membranes, *L*_x_ calculated using the Hagen–Poiseuille equation tended to overestimate the true axial conductance of a leaf. To understand how changes in *L*_x_ would lead to changes in *K*_leaf_ and *K*_r_, the model was run with values for *K*_x_ ranging from 100 to 5% of its value calculated using Eq. 1. As long as *K*_x_ was greater than 40% of its initial value, *K*_leaf_ was equal to or greater than 90% of its maximum value (Figure 5A). Not until *K*_x_ was reduced to 5% of its value did *K*_leaf_ decrease by half.

Another way to analyze the effect of reductions in *K*_x_ is to consider how changing *K*_x_ (=1/*R*_x_) affected the relative leaf resistances. In terms of resistance, *R*_x_ was initially low in comparison to *R*_r_ (Figure 5B). This ratio increased to 0.45 when *K*_x_ was decreased to 50% of its maximum predicted value, and the ratio was about 1.5 when *K*_x_ was decreased to 20%. Below 20% of maximum *K*_x_, *R*_x_ sharply increased relative to *R*_r_. In terms of radial resistance, the ratio of *R*_r_ to *R*_leaf_ was approximately 0.9 or larger as long as *K*_x_ was 50% or more of its maximum value (Figure 5C). Even when *K*_x_ was decreased to 20%, *R*_r_ equaled approximately 0.8 of *R*_leaf_.

According to the model used to calculate *K*_r_ and *K*_leaf_, the effective leaf length *l*′ is an index of how far along a leaf water can be transported and transpired depending on leaf size, conductances, and the gradient in water potential. Based on conductances in Figure 4, *l*′ was initially 94 ± 1% of the actual leaf length *l* (Figure 5D). As long as *K*_x_ was 60% or more of its maximum predicted value, the ratio of *l*′ to *l* was 0.9 or greater, but *l*′ decreased to about half of *l* when *K*_x_ was 10% of its maximum value.

## Discussion

As predicted on the basis of low photosynthetic rates, shade tolerance, and long leaf lifespan, *G. lingulata* had a low leaf hydraulic conductance (*K*_leaf_) compared with most other angiosperms, indeed, even compared with most ferns and gymnosperms (Brodribb et al., 2007). Like most tank bromeliads, *G. lingulata* is conservative with respect to photosynthesis and water use. Maximum rates of net CO_2_ uptake (*A*_max_), which show close correlation with *K*_leaf_ in a wide range of species (Brodribb et al., 2007), are also low for *G. lingulata*, with *A*_max_ measured at approximately 1.6 μmol m^−2^ s^−1^ in both field (Griffiths et al., 1986) and laboratory (Maxwell, 2002). This value is even lower than the average *A*_max_ measured for epiphytic ferns at La Selva Biological Station in Costa Rica (Watkins et al., 2010). In addition, *K*_leaf_ is negatively correlated with leaf lifespan, at least on a dry mass basis (Simonin et al., 2012), and adult leaves of tank bromeliads such as *G. lingulata* are usually retained for more than a year (Meisner and Zotz, 2012). Whether low *K*_leaf_ would constrain photosynthetic rates or vice versa is unknown, but both rates are consistent with low growth rates and limited resources in a highly changeable environment.

With respect to water use traits measured in this study, stomatal conductance to water vapor *g*_s_ was low for plants of *G. lingulata* at La Selva and in a glasshouse at Occidental College, averaging less than 30 mmol m^−2^ s^−1^. This value is somewhat higher than that previously measured for *G. lingulata* in the laboratory (10 mmol m^−2^ s^−1^; Maxwell, 2002) yet is still in keeping with its low photosynthetic rate. Moreover, low *g*_s_ reflected the low stomatal density for *G. lingulata* in the field and glasshouse, which was comparable to stomatal densities measured for its congener *G. monostachia* (Freschi et al., 2009), for tank-forming *Tillandsia* species (Reyes-Garcia et al., 2008), and for desert succulents (Larcher, 2003). Stomatal density for *G. lingulata* was more than 10 times lower than the average reported for a group of shade-tolerant tropical trees (Sack et al., 2005). Assuming that stomatal pore sizes are not unusually large in leaves of *G. lingulata*, its low *K*_leaf_ was congruent with stomatal traits associated with extremely conservative water use. Moreover, low porometer readings for plants at La Selva and in the glasshouse indicate that stomatal closure was a pervasive response, even when tank water was present.

The large airspaces subtending the stomates in leaf blades of *G. lingulata* could also be associated with conservation of water in that the increased distance between the guard cells and internal cell surfaces would decrease the rate of diffusion (Nobel, 2009). However, the size of the airspaces far exceeds what would be predicted on the basis of water conservation alone (Pickard, 1982). The location of the principal photosynthetic mesophyll in the center of the leaf suggests that an alternative role for the airspaces is to facilitate the uptake of CO_2_, which diffuses more rapidly through air than through cells (Pickard, 1982). Particularly when stomates are closed, the internal airspaces could serve as reservoirs of CO_2_, similar to the tank’s role as reservoir for water. The central location of the mesophyll surrounding the leaf veins in itself may reduce water loss from the mesophyll as well as reduce the distance that photosynthate must travel to the phloem. In addition, the relatively high volume of internal airspace may also improve the carbon assimilation and water use efficiency of leaves of *G*. *lingulata*, as discussed for a group of Mediterranean tree species (Mediavilla et al., 2001).

The distances that water and CO_2_ travel within leaves have been quantified in a number of ways, including the distance between veins (*D*_iv_), the distance between the center of a vein and the epidermis (*D*_epi_), and the distance along the cellular pathway between the center of a vein and the closest stomate (*D*_mes_). According to a recent study of several species from a wide range of habitats and an artificial leaf made to mimic properties of water flow through leaves, *D*_epi_ (about half of leaf thickness) and *D*_iv_ are related such that *D*_iv_ = 1.08× *D*_epi_ (Noblin et al., 2008). It is reasonable that the closer the evaporative surface, the more closely spaced that veins would have to be to meet transpirational demand. Using values from Table 2, *D*_iv_ = ×2.0 *D*_epi_ for leaf blades and 1.7 for the tank region, suggesting that interveinal distance for leaves of *G. lingulata* may limit water flow between the vasculature and the leaf surface. However, *D*_mes_ tells a different story, with its mean value of 181 for leaf blades of *G. lingulata* comparable to that for angiosperms with much higher rates of *K*_leaf_ (Brodribb et al., 2007). Differences in the way that *D*_mes_ was measured in this study (by tracing cell contours) and by Brodribb et al. (calculated from the numbers and shapes of more tightly packed cells) may account for the lower than predicted *D*_mes_ for *G. lingulata*. In any case, the significance of *D*_mes_ may in part depend on whether water travels predominantly through cell walls or through the symplast or transcellular pathways.

Using dyes to trace the movement of water through plant tissues has a long but somewhat ambiguous history. The path of the apoplastic dye basic fuchsin used in this study did suggest that water taken up by cut leaf blades traveled through the xylem and exited tracheids through bundle sheath cells and through nearby cell walls (Figure 3A). Radial discontinuities in the lignified, suberized cell wall of bundle sheath cells indicated the presence of plasmodesmata, which tended to stain deeply, suggesting that water crossed cells of the bundle sheath through the symplast. Thus, the bundle sheath was not radially impermeable, but it may have slowed the passage of water. Furthermore, the involvement of the symplast in bundle sheath cells suggests a possible role for aquaporins in regulating water movement into and out of the veins (Shatil-Cohen et al., 2011). The movement of sulforhodamine G through the cut leaf blade was similar to that of basic fuchsin. Since both dyes are thought to be confined to the apoplast, staining patterns in the leaf blade did not clarify the role of the symplast in water flow, at least in the leaf blade.

In contrast to the leaf blade, the tank region of the leaf permitted sulforhodamine G to move from the leaf surface through both the symplast and apoplast to the veins (Figure 3B). Because the dye did not appear in all cells (those cut so that vacuole contents were lost) or in the large central airspaces, the staining did not seem to be an artifact. Thus, both sulforhodamine G and water moved across cell membranes as well as through cell walls, at least in the tank region, possibly implying an easier radial pathway for water into the leaf than out for *G. lingulata*. Another implication is that sulforhodamine G is not strictly an apoplastic dye. The tank region had other anatomical traits associated with more rapid water uptake than for the leaf blade, specifically, a significantly greater density of epidermal trichomes and greater tracheid diameters(Table 2). Trichomes can be considered roughly analogous to root hairs with regard to water absorption, and the greater tracheid diameter for the tank can be considered functionally equivalent to the greater diameter of xylem conduits in roots than in shoots (Tyree and Zimmermann, 2002; North, 2004). With respect to both axial and radial pathways, the tank region of *G. lingulata* leaves was suited to greater hydraulic conductance than the blade.

Traits related to the axial pathway for water movement include the density of veins (*D*_v_) as well as the diameter of tracheids and the number of vascular bundles. Leaves of *G. lingulata* had low values of *D*_v_, particularly in the tank region, compared to most other species. Specifically, values for *D*_v_ of about 4.8 mm mm^−2^ in the leaf blade and 3.8 mm mm^−2^ in the tank region place *G. lingulata* in the bottom third of a large group of extant species and fossil inventories, ranking with basal eudicots and Magnoliids (Brodribb and Feild, 2010). For the bottom third of species, low *D*_v_ was correlated with low photosynthetic capacity, comparable to (or even higher than) rates previously measured for *G. lingulata*. Although *D*_v_ did not differ between plants from La Selva or the glasshouse, the percent of cross-veins was higher for the former (14%) than for the latter (10%). Thus, not taking into account the cross-veins in calculating *K*_x_ was slightly more important for plants from La Selva than from the glasshouse, but was probably not a substantial source of error in either case.

In keeping with other anatomical traits, tracheid diameters for leaves of *G. lingulata*, with a mean for leaf blades of approximately 6.5 μm, were at the low end of the range reported for xylem conduits in the leaves of other species. For example, conduit diameters are about 20 μm in the stipes of fern epiphytes (Watkins et al., 2010), 7-22 μm for the protoxylem and 22 μm for the metaxylem in blades of the grass *Festuca arundinacea* (Martre and Durand, 2001), 12-30 μm for leaves of the tropical conifer *Podocarpus grayi* (Brodribb and Holbrook, 2005), and 10-15 μm for secondary veins in leaves of 10 species of temperate oaks (Coomes et al., 2008). Smaller conduit sizes are reported only for minor veins, e.g., about 4-5 μm for fourth order and higher veins in leaves of walnut and laurel (Cochard et al., 2004). The mean maximum tracheid diameter of 11.7 μm for *G. lingulata*, measured for two to three tracheids in every fourth vein, is comparable to values for secondary veins in leaves of walnut and laurel (Cochard et al., 2004). Thus, there could be a division of labor for veins in *G. lingulata*, those with larger diameter tracheids predominating in axial flow and those with smaller tracheids leaking water in a radial direction (Canny, 1991).

The leaky cable model developed for the simple, strap-shaped leaf typical for tank bromeliads allowed assessment of the relative contributions of axial (*K*_x_) and radial (*K*_r_) conductances to total leaf hydraulic conductance (*K*_leaf_) for *G. lingulata*. Expressed on a leaf area basis, *K*_x_ was the largest conductance of the three (Figure 4); put another way, 1/*K*_x_, or *R*_x_, was the smallest component of total leaf resistance (*R*_leaf_). Specifically, the ratio of *R*_x_/*R*_leaf_ was 0.18, and the ratio of *R*_x_ to radial resistance (*R*_r_) was 0.19. An important caveat is that the model used values for *K*_x_ that were calculated using the Hagen–Poiseuille equation, which is known to overestimate the true axial conductance in almost all cases (Tyree and Zimmermann, 2002). In a careful analysis of the relationship between measured axial conductance and that predicted using Hagen–Poiseuille for leaf blades of *F. arundinacea*, the ratio of predicted/measured conductance was about 0.20 (Martre et al., 2001). This ratio was considered to be about 0.12–0.28 for leaves of *Laurus nobilis* (Cochard et al., 2004). Multiplying *K*_x_ by a factor of 0.2 and using this value in the model resulted in the axial resistance nearly equaling the total leaf resistance. When *K*_x_ was 0.2 of its calculated value, *R*_r_/*R*_leaf_ was 0.7. The same reduction in *K*_x_ reduced the effective leaf length to about 0.7× the actual length along which water could be supplied. Thus, if the relationship between predicted and true axial hydraulic conductance for *G. lingulata* is similar to that for fescue and laurel, then the radial resistance accounted for about 70% of the total leaf hydraulic resistance.

Certain methods and assumptions made by the model used to calculate *K*_r_ could be modified to improve future analyses. Foremost would be the use of real (or measured) instead of predicted *K*_x_. The large airspaces in leaves of *G. lingulata* ruled out applying positive or negative pressure to force water through the cut end of the leaf blade, but experimentation with plugging such channels (e.g., with dental impression material) is ongoing. A more realistic treatment of leaf shape should be incorporated into the model to allow for taper at the leaf apex. Two other concerns are that leaf capacitance was not considered, and leaf water potential was treated as invariant along the length of the leaf. These are likely sources of error, as suggested by the amount of hydrenchyma (water storage parenchyma) in leaves of *G. lingulata* and evidence that Ψ_leaf_ decreased by as much as 0.2 MPa between the base of the leaf blade and the apical region (data not shown). However, Ψ_leaf_ was similar to values measured for plants of *G*. *lingulata* in the field during the rainy season (Smith et al., 1985) and thus is a realistic representation of the driving force for water uptake from the tank. More research is needed to be able to predict changes in Ψ_leaf_ and *K*_leaf_ during dry periods when tank water is not available.

In conclusion, the extremely low leaf hydraulic conductance for the tank bromeliad *G. lingulata* was the product of structural features in both the axial and radial pathways for water flow in the leaf. Narrow tracheids and infrequent veins led to low axial conductance, and bundle sheath cells with thick lignified, suberized call walls restricted water flow in the radial direction. Notably, the tank region had larger tracheids than did the leaf blade and was more permeable to tracer dyes, indicating that supply of water from the tank should not be a hydraulic constraint for *G. lingulata*. The model used to calculate radial conductance indicated that radial resistance represented about 70% of the total leaf hydraulic resistance when axial conductance was 20% of the predicted value; axial and radial resistances were about equal when *K*_x_ was 25% of that predicted. Overall, leaf hydraulic conductance for *G. lingulata* and its associated anatomical features were in keeping with an extremely conservative use of water, a limited and variable resource for epiphytic tank bromeliads.

## Conflict of Interest Statement

The authors declare that the research was conducted in the absence of any commercial or financial relationships that could be construed as a potential conflict of interest.

## Footnotes

^1^http://rsb.info.nih.gov/ij/

^2^http://www.tropiflora.com/
